# Quantitative cardiac autonomic outcomes of hydrotherapy in women during the first stage of labor

**DOI:** 10.3389/fmed.2022.987636

**Published:** 2023-01-03

**Authors:** Raquel Aparecida Dias, Cláudia de Faria Cardoso, Rym Ghimouz, Daniel Alessander Nono, José Antônio Silva, Juan Acuna, Ovidiu Constantin Baltatu, Luciana Aparecida Campos

**Affiliations:** ^1^Center of Innovation, Technology and Education (CITE) at Anhembi Morumbi University—Anima Institute, São José dos Campos Technology Park, São José dos Campos, Brazil; ^2^Fatima College of Health Sciences, Abu Dhabi, United Arab Emirates; ^3^Center for Special Technologies, National Institute for Space Research (INPE), São José dos Campos, Brazil; ^4^Universidade Nove de Julho, São Paulo, Brazil; ^5^Department of Public Health and Epidemiology, College of Medicine and Health Sciences, Khalifa University, Abu Dhabi, United Arab Emirates

**Keywords:** heart rate, autonomic nervous system, anxiety, childbirth, biomarkers

## Abstract

**Introduction:**

Most hydrotherapy studies during childbirth report findings related to pain using a widespread set of subjective measures. In this study, ECG biomarkers as quantitative cardiac autonomic outcomes were used to assess the effects of warm shower hydrotherapy on laboring women during the first stage of labor.

**Methods:**

This was a prospective single-blind cohort study on stage I delivering women. Their cardiac autonomic function was assessed using heart rate variability (HRV) measures during a deep breathing test using point-of-care testing comprised of an HRV scanner system with wireless ECG enabling real-time data analysis and visualization. Labor pain and anxiety were assessed using the Visual Analog Scale for Pain (VASP) and the Beck Anxiety Inventory (BAI). A total of 105 pregnant women in the first stage of labor who received warm shower hydrotherapy, intravenous analgesia (scopolamine + sodium dipyrone), or spinal anesthetic (bupivacaine + morphine) were enrolled.

**Results:**

In women during the first stage of labor, parasympathetic modulation reflected through RMSSD (root mean square of successive RR interval differences) was significantly reduced by hydrotherapy and intravenous analgesia (before vs. after mean rank diff. 35.73 and 65.93, respectively, *p* < 0.05). Overall HRV (SDNN, standard deviation of RR intervals) was significantly decreased only by intravenous analgesia (before vs. after mean rank diff. 65.43, *p* < 0.001). Mean heart rate was significantly increased by intravenous analgesia, while spinal anesthesia reduced it, and hydrotherapy did not alter it (before vs. after mean rank diff. –49.35*, 70.38*, –24.20*^NS^*, respectively, *p < 0.05, *^NS^* not significant).

**Conclusion:**

This study demonstrates that warm shower therapy may impact the sympathovagal balance *via* parasympathetic withdrawal in women during the initial stage of labor. The findings of this study provide quantitative support for using warm shower hydrotherapy during labor *via* point-of-care testing. The dependability of hydrotherapy as a non-pharmacological treatment is linked to the completion of more clinical research demonstrating quantitative evidence *via* outcome biomarkers to support indications on stress and birth progress.

## Introduction

Hydrotherapy is a traditional medical and health intervention used as an alternative to spinal anesthesia in women during the first stage of labor to decrease parturient anxiety and promote relaxation (1). The American College of Nurse-Midwives (2017) (2) and the American College of Obstetricians and Gynecologists (2016) (3) recently published practice guidelines for the use of immersion/bathing during labor and birth. Hydrotherapy, including water immersion and a warm shower during the first stage of labor, may be associated with decreased labor and reduced use of spinal and epidural analgesia and may be provided to healthy women with uncomplicated pregnancies between 37 0/7 weeks and 41 6/7 weeks of gestation (4). Studies show the effects of hydrotherapy in reducing maternal anxiety (5, 6).

As a hydrotherapy method, a warm shower during the first stage of labor is in an incipient phase of adoption by maternity clinics (6). A warm shower is a midwife-centered intervention that can be used in most hospitals. Such intervention has no reported side effects for the mother or her infant, and it also reduces the need for epidural anesthesia, episiotomy, and instrumental delivery (7). Therapeutic showering proved beneficial for alleviating pain, discomfort, anxiety, and tension while promoting relaxation and facilitating labor (6). A systematic review of 3,243 women revealed that showering decreases the severity of pain in laboring women, reduces the requirement for pharmaceutical analgesia, and shortens the first phase of labor (8). Pregnant women generally rate the experience of a warm shower during labor favorably (6, 9). In Brazil, whether nulliparous or multiparous, pregnant women are well aware of the pain-relieving effects of taking warm showers during childbirth (10). It has been used successfully in Brazil to alleviate labor pain during the first stage of the labor (11).

Labor induces autonomic responses that control uterine contractions and regional blood flow. Previous research on sympathovagal balance during the first stage of labor was primarily aimed at assessing cardiac autonomic modulation during uterine contractions (12, 13). During labor, there is an increase in vagally mediated activity, which could be due to oxytocin-induced cholinergic pathway activation or the anti-inflammatory cholinergic response (13). The association of anxiety with the autonomic nervous system is well-known (14). Data on heart rate variability (HRV) as measures for anxiety levels indicate that fear of childbirth was associated with low parasympathetic activity among low-risk pregnant women (15). Few studies have investigated the relationship between psychological stress and autonomic nervous activity during parturition or even during pregnancy (16–18).

In summary, hydrotherapy has been associated with potentially beneficial neuroendocrine stress changes promoting adaptation to stress and anxiety in women during childbirth (19, 20). However, most hydrotherapy studies report findings related to pain, stress, and anxiety using subjective markers. This can hamper the process of justifying the integration of this potentially beneficial intervention into the delivery management strategies. Quantitative evidence for the use of hydrotherapy in women during the first stage of labor is needed.

In the present study, we explored the hypothesis that warm shower hydrotherapy has an effect on the cardiac autonomic nervous system. For this aim, we quantified the effect of a warm shower on the cardiac autonomic nervous system and anxiety in laboring women during the first stage. We did not pose a directional hypothesis about the impact outcomes due to a limited number of studies on autonomic nervous system responses to tasks that elicit a range of emotions, such as labor expectations (13, 21).

## Materials and methods

### Study design and participants

This prospective cohort study was conducted at Hospital São Francisco de Assis, Jacareí/Sao Paulo, Brazil. The protocol was approved by Anhembi Morumbi University Ethical Committee (CAAE: 15787719.50000.5492). The study was carried out following the resolutions 466/2012 and 340/2004 of the National Health Council (Ministry of Health) for research on human subjects and with international medical ethics guidelines (Geneva Declaration, International Code of Medical Ethics, 1948, amended 1983). After being admitted to the maternity hospital for delivery, all study participants provided written informed consent to participate in the study.

This study recruited women admitted for delivery at the Maternity Hospital São Francisco de Assis, Jacareí, Brazil. Inclusion criteria were adult (18 years or more) pregnant women with a gestational age of more than 37 weeks, who were registered with the hospital prenatal care program no later than 16th week of gestation, with singleton pregnancy in cephalic presentation, no previous cesarean, cervical dilation between 3 and 7 cm at admission, receiving no pain medication, without any aggregated pathology and at low risk for obstetrical complications. Participation in the hospital prenatal care program associated with the Unified Health System (SUS, Sistema Único de Saúde) was defined as a pregnant woman initiating prenatal care visits no later than the 16th week of pregnancy and with an adequate number of appointments as defined by the standard of care (22). According to governmental guidelines (22), information about pain management was provided as part of the prenatal education program. Potential participants were identified by nurses and obstetrical care providers. The consent process was carefully conducted before the onset of labor through effective uterine contractions (23) to enhance women’s autonomy (24). Exclusion criteria were arterial blood pressure < 90/60 mmHg, previous regional or opioid analgesia, alcohol or drug abuse or addiction, non-pregnant subjects, and pre-pregnancy comorbidities, such as chronic arterial hypertension, and diabetes mellitus.

At the start of the research, a snowball sampling was used to select an initial group of pregnant women who signed the informed consent before the onset of labor. Five pregnant women signed the informed consent form and opted out of the study. A convenience sample of eligible women was selected to differentiate two groups of pharmacological treatment (spinal anesthesia and intravenous analgesia). Three study groups of eligible women who signed the informed consent were formed: (1) experimental, which received a warm shower, *n* = 45 women; (2) active comparator, which received intravenous analgesia, *n* = 30 women; (3) active comparator, which received spinal anesthesia, *n* = 30 women. Warm shower hydrotherapy was for 30–45 min, with temperature maintained at around 37 degrees Celsius. Spinal anesthesia was with hyperbaric bupivacaine plus morphine (2.5 ml Marcain Heavy 0.1 mg Dimorf^®^) (25) analgesic or (26). Intravenous analgesia was with scopolamine plus sodium dipyrone [5 ml scopolamine butylbromide + sodium dipyrone (Buscopan) + 2 ml Metoclopramime (Plasil) + 10 ml 25% glucose + 100 ml 0.9% physiological saline]. The investigated outcome measures were before and after the interventions (warm shower, intravenous analgesia, or spinal anesthesia). The data were anonymized and handled according to the Brazilian General Data Protection Law (27). The interventions were coded, and the statistician remained blinded to the coding allocation until the analysis was complete (28).

### Outcome measures

#### Point-of-care quantitative autonomic testing—deep breathing test

**FIGURE 1:**
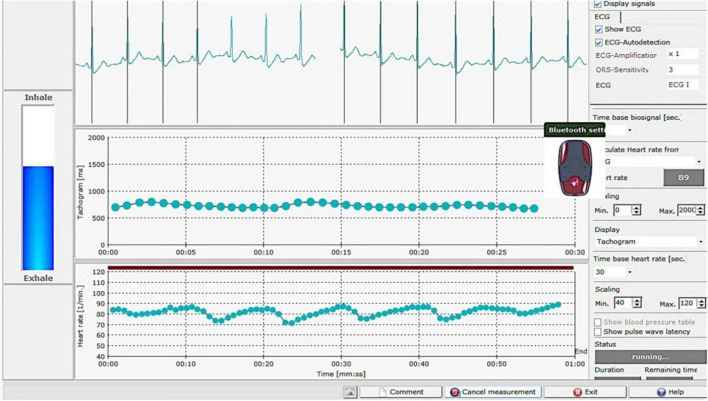
Screenshot of a deep breathing test performed with the HRV-scanner software, which employs a respiration pacer (bar on the left side of the screen) to assess and analyze heart rate variability in response to deep breathing.

A quantitative assessment of cardiac autonomic function was conducted on stage I delivering women during a deep breathing exercise, as previously reported (29, 30). The HRV deep breathing test was performed using a wireless sensor device for monitoring single channel ECG and HRV and HRV-scanner software (Mega Electronics, Finland), which utilizes a respiration pacer to assess and analyze the variability in the heart rate in response to deep breathing (Figure 1). This autonomic nervous system activity assay using the wireless ECG system meets the logistical requirements for a point-of-care test (31). The study participants were instructed to breathe in time to the computer-generated respiration pacer bar set at a respiratory rate of six breaths per minute (left side of the screen, Figure 1) and to freely adjust their tidal volume to a comfortable level to maintain minute ventilation. The cardiac autonomic function of women during the first stage of labor was examined by analyzing the R-R intervals during a 1-min recording over six respiratory cycles. The HRV-Scanner detects artifacts automatically by calculating a quality parameter called “RSA Quality.” This parameter describes the deviation of the heart rate trace from an idealized sinus. The deep breathing test was performed again if the value is less than 50%. After 1 min of high-quality recording, the deep breathing test will automatically stop recording. Following the deep breathing test measurement, the software computes the HRV parameters: SDNN (ms, standard deviation of normal R-R wave intervals), RMSSD (ms, square root of the mean of the number of squares of variations between adjacent normal R wave intervals), coefficient of variation (CV, standard deviation to mean ratio), SD1 (Poincaré plot standard deviation perpendicular to the line of identity), SD2 (Poincaré plot standard deviation along the line of identity), and E/I (ratio of exhalation vs. inhalation).

#### Anxiety and pain evaluation

Self-perceived symptoms of anxiety were assessed with the Beck Anxiety Inventory (BAI) (32), which was constructed to avoid confounding with depression (33). The BAI is a scale with good psychometric properties that can be used in situations where physiological symptoms are essential, such as during childbirth. It has been validated in Brazilian subjects (34). It consists of 21 items, each of which is a statement describing anxiety symptoms that participants must rate on a Likert scale of 4 points. According to the total score ranging from 0 to 63, anxiety levels are categorized as follows: minimal anxiety (0–7), mild anxiety (8–15), moderate anxiety (16–25), and severe anxiety (26–63) (34, 35). After the interventions, the study participants were asked to report if the intervention led to the relief of anxiety or not. Pain levels were evaluated before and after interventions using the Visual Analog Scale of Pain (VAS), a tool previously used to assess various pain conditions, including childbirth (36). The VAS pain score was from 1 to 3: mild, 4–6: moderate to 7–10: severe (37). While the visual analog scale could be used both before and after the interventions due to its demonstrated sensitivity to hourly changes in the pain (38), the BAI could only be used before the interventions due to its demonstrated test-retest reliability only at days/weeks intervals (39, 40). This is why the anxiety after the intervention was reported as a percentage of women who reported anxiety relief compared to the moment before the intervention.

Parturients were given enough time to choose the anesthetic method provided they met the inclusion and exclusion criteria and fell under risk classification category 4 (category 4: No maternal or fetal compromise and can be done at a time to suit the woman and maternity services) (41). The study protocol was as follows: (1) before interventions, the parturients responded to VAS and BAI, and then they underwent the 1st deep breathing test; (2) the interventions were warm shower hydrotherapy (30–45 min); intravenous analgesia, or spinal anesthesia; (3) after interventions (immediately after the warm shower hydrotherapy, or immediately after the analgesia or anesthesia became effective), the parturients responded to VAS and the question whether or not there was a reduction in anxiety, and then they underwent the 2nd deep breathing test, which was between 30 and 45 min from the 1st test.

### Statistical analysis

Data were tested for normality distribution using the D’Agostino-Pearson normality test (42) and Kolmogorov-Smirnov test with Dallal-Wilkinson-Lilliefors’ *P*-value (43). Differences between study groups were assessed with the Kruskal-Wallis test, followed by Dunn’s test correction for multiple comparisons using statistical hypothesis testing. These multiple comparisons were of mean ranks of preselected data pairs (hydrotherapy, intravenous analgesia, and spinal anesthesia groups): before and after the interventions. These statistical analyses were carried out using GraphPad Prism version 8.1.2 for Mac OS X, GraphPad Software, La Jolla California USA.^1^ Differences were considered significant when the Type I error probability was lower than 5% (*p* < 0.05). Proportions comparisons were performed using the MedCalc^®^ Statistical Software version 19.5.3 (MedCalc Software Ltd., Ostend, Belgium; 2020), which employs the “N-1” Chi-squared test as recommended by Campbell (44) and Richardson (45).

## Results

### Maternal characteristics

During the study period, 143 eligible women were invited to participate; 110 agreed to participate and signed the informed consent; 5 women opted out after signing the consent, resulting in 105 women being included in the study. Participants’ maternal ages were 25.20 (23.95–26.50) (geometric mean with 95% CI), and their gestational ages were 39.10 (38.86–39.34) (geometric mean with 95% CI). Ninety percent of the study participants had completed secondary school. Of them, 56% were nulliparous, and 44% were multiparous. None of the study participants had a prior cesarean delivery. Fifty-two and four-tenths (52.4%) had no associated pathologies, 21.9% had gestational diabetes or hypertension, and 25.7% had pyelonephritis or urinary tract infection. Forty-five women received warm shower hydrotherapy (52% nulliparous and 48% multiparous), 30 received spinal anesthesia (60% nulliparous and 40% multiparous), and 30 received intravenous analgesia (50% nulliparous and 50% multiparous). The time between the first HRV deep breathing test and birth was 170.7 (145.9–199.9). The percentage of cesarian delivery among the study groups was: (1) 29% in the warm shower group, (2) 30% in the intravenous analgesia group, and (3) 100% women in the spinal anesthesia group. The newborns were 87.6% without complications, 6.7% with respiratory distress syndrome, 3.8% with meconium aspiration syndrome, and 1.9% with neonatal bradycardia.

### Quantitative autonomic measures

Descriptive statistics of HRV indexes from deep breathing tests are presented in Table 1. Since RMSSD with Poincaré SD1 (46, 47) and SDNN with Poincaré SD2 (48) are analog metrics of HRV, only RMSSD and SDNN measures were used for further intergroup comparisons.

**TABLE 1:** Effect of hydrotherapy (HydroTx), intravenous analgesia (IV ANALG), spinal anesthesia (Spinal ANES) on heart rate variability indexes from deep breathing test.

HRV index	HydroTx		IV ANALG		Spinal ANES	
	Before	After	Before	After	Before	After
Mean heart rate	81.87 (79.59–86.03)	86.18 (84.83–91.85)	82.26 (79.63–90.16)	102.10 (93.16–106.30)**	84.83 (80.30–89.22)	69.06 (66.61–72.97)**
RMSSD (ms)	42.42 (43.23–59.82)	30.69 (29.3–38.01)*	46.19 (33.11–57.67)	9.77 (8.96–33.15)**	40.65 (35.19–51.12)	40.65 (35.19–51.12)
SD1	29.28 (31.06–43.97)	22.15 (21.62–28.37)*	32.67 (23.41–40.78)	6.91 (6.34–23.44)**	28.74 (24.88–36.15)	42.76 (35.37–52.48)
SDNN (ms)	70.96 (68.75–87.68)	64.85 (56.09–72.5)	68.41 (56.2–84.87)	22.6 (22.89–47.01)**	73.4 (62.10–82.32)	85.94 (69.07–91.48)
SD2	95.58 (92.04–115.7)	87.98 (77.7–99.66)	89.45 (75.46–112.70)	31.53 (31.61–61.6)**	99.90 (83.14–110.60)	112.30 (90.28–118.10)
CV (ms)	9.44 (9.32–11.47)	9.11 (8.26–10.28)	9.10 (7.72–10.84)	3.99 (3.74–6.53)**	10.50 (8.66–11.22)	9.28 (7.89–10.19)
E/I	1.29 (1.28–1.37)	1.23 (1.22–1.30)	1.28 (1.23–1.35)	1.07 (1.07–1.18)**	1.29 (1.24–1.33)	1.27 (1.23–1.34)

RMSSD, root-mean-square of differences between adjacent normal RR intervals; SD1, Poincaré plot standard deviation perpendicular to the line of identity; SDNN, standard deviation of all normal RR intervals; SD2, Poincaré plot standard deviation along the line of identity; CV (Coefficient of Variation), standard deviation (of the HRV data) divided by the mean HRV value and expressed as a percentage; E/I, ratio of exhalation vs. inhalation. The Kruskal-Wallis test and Dunn’s test correction for mean ranks comparisons (before and after) were used to analyze differences between study groups; data are presented as median values with 95% confidence intervals; * < 0.05, ** < 0.001.

**FIGURE 2:**
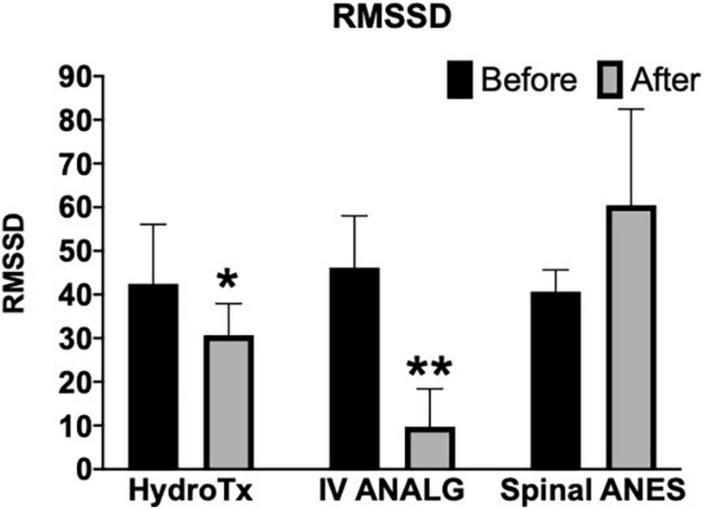
Effect of hydrotherapy (HydroTx), intravenous analgesia (IV ANALG), and spinal anesthesia (Spinal ANES) on cardiac parasympathetic modulation (RMSSD, which is an identical metric with Poincaré plot SD1). Values are median with a 95% confidence interval. Kruskal-Wallis test followed by Dunn’s test correction for mean ranks comparisons (before and after) using statistical hypothesis testing; *p < 0.05, **p < 0.005.

**FIGURE 3:**
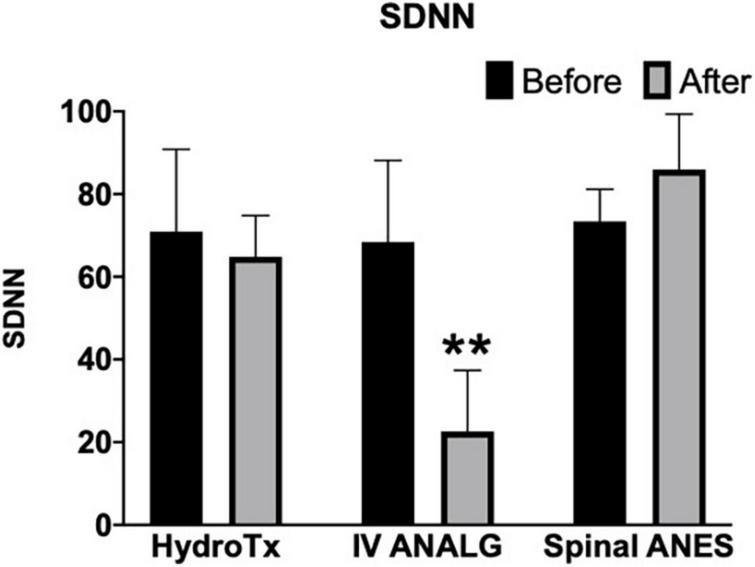
Effect of hydrotherapy (HydroTx), intravenous analgesia (IV ANALG), spinal anesthesia (Spinal ANES) on overall heart rate variability (SDNN). SDNN (identical metric with Poincaré plot SD2) reflects sympathetic and parasympathetic modulation. Kruskal-Wallis test followed by Dunn’s test correction for mean ranks comparisons (before and after) using statistical hypothesis testing; **p < 0.005.

**FIGURE 4:**
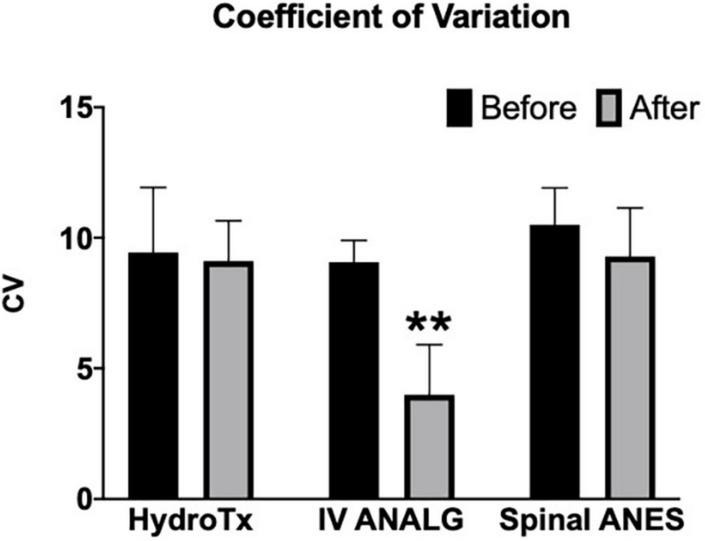
Effect of hydrotherapy (HydroTx), intravenous analgesia (IV ANALG), and spinal anesthesia (Spinal ANES) on heart rate coefficient of variation (CV) reflecting overall cardiac variability. Values are median with a 95% confidence interval. Kruskal-Wallis test followed by Dunn’s test correction for mean ranks comparisons (before and after) using statistical hypothesis testing; **p < 0.005.

**FIGURE 5:**
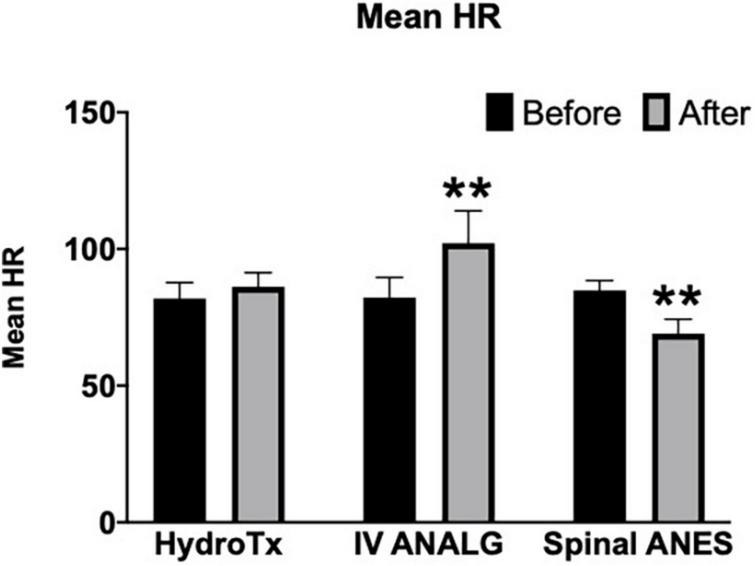
Effect of hydrotherapy (HydroTx), intravenous analgesia (IV ANALG), spinal anesthesia (Spinal ANES) on Mean Heart Rate—mean ranks comparisons. Values are median with a 95% confidence interval. Kruskal-Wallis test followed by Dunn’s test correction for mean ranks comparisons (before and after) using statistical hypothesis testing; **p < 0.005.

Parasympathetic modulation reflected through RMSSD was significantly reduced by warm shower hydrotherapy and intravenous analgesia (after vs. before mean rank diff. –35.73 and –65.93, respectively, *p* < 0.05) (Figure 2). Overall, HRV (SDNN and CV) was significantly decreased only by intravenous analgesia (after vs. before mean rank diff. –65.43, *p* < 0.001) (Figures 3, 4). Mean heart rate was significantly increased by intravenous analgesia, while spinal anesthesia decreased with no effect of hydrotherapy (after vs. before mean rank diff. 49.35*, –70.38^**^, 24.20^NS^, respectively, *p < 0.05, ^**^p < 0.001, ^NS^ not significant) (Figure 5).

### Anxiety and pain outcome measures

The anxiety levels assessed with the BAI were mild in a majority of 65.7% of women, followed by 20.0% of women with moderate anxiety and 14.3% of women with minimal anxiety. A significantly higher percentage of women reported anxiety relief from warm shower hydrotherapy (87.5%) during the first stage of labor than from intravenous analgesia (40%, Chi-squared significance of *p* < 0.05), which was comparable to spinal anesthesia (86.7%).

Before interventions, there were no differences in reported pain between the study groups (median with 95% confidence interval): 7.0 (5.75–6.88) in the hydrotherapy group, 7.5 (5.03–6.97) in the intravenous analgesia group, and 7.0 (5.85–7.36) in the spinal anesthesia group. While intravenous analgesia and spinal anesthesia eliminated pain in the study participants, a warm shower significantly reduced pain perception from 7 (5.75 to 6.88) to 5 (4.33 to 5.05), *p* < 0.001.

There were no significant associations between HRV indexes, maternal age, pregnancy age, level of education, parity, anxiety and pain levels, cervical dilation, or associated pathologies using univariate analysis, as these theories were not tested, and therefore the research was not powered to investigate and identify such variations.

## Discussion

Using autonomic function biomarkers, this study demonstrated that warm shower hydrotherapy affects cardiac autonomic function *via* parasympathetic withdrawal in women during the first stage of labor. These findings provide quantitative evidence for warm shower hydrotherapy as a non-pharmacological approach during the first stage of labor to decrease parturient anxiety and promote relaxation.

Non-pharmacological approaches to inducing labor and managing pain in women during childbirth are widely used, but there is still a paucity of scientific evidence to support this (49). Senses of Birth (SoB) is a health education intervention in Brazil that seeks to minimize unnecessary medicalization for women during childbirth by offering information on evidence-based practices (EBP) (50). A recent integrative review has listed studies on non-pharmacological therapies with immediate effects in labor, such as massage, hot baths, Swiss ball, breathing techniques, and supportive care (51). Rates of hydrotherapy use differ widely from country to country (4, 52). As a hydrotherapy method, a warm shower during the first stage of labor has been implemented as a routine procedure for parturition at the maternity of Hospital São Francisco de Assis (Jacareí/São Paulo, Brazil) according to the recommendations and guidelines of the governmental Unified Health System (SUS, Sistema Único de Saúde) (53). Research conducted in two Brazilian hospitals found that hydrotherapy was clinically beneficial to the progression of labor, increasing the frequency of uterine contractions and fetal heart rate (54). This study’s results agree with a recent randomized controlled trial, where warm showers as an easy-to-deploy, non-pharmacological approach could reduce pain during the first labor stage (36). Inconsistent associations between age, parity, stress, and anxiety have been reported (55). The present study found no correlations between HRV indexes and anxiety. This could be due to the BAI ’s inability to be used immediately after anesthesia, as well as a lack of evidence demonstrating its short (hours) test-retest reliability. A shorter, more pragmatic screening tool that could allow test-retest at short time intervals has been demanded to evaluate the fear and anxiety of childbirth (55).

According to this study’s findings, using warm shower therapy during the first stages of labor may modulate the sympathovagal balance in women by inducing a parasympathetic withdrawal response. Parturition involves the coordination of many neuro-immune-endocrine interactions, including activation of the autonomic function (56). Previous studies show that the autonomic nervous system is modulated during parturition, with parasympathetic involvement linked either to the releases of oxytocin or to the activation of the cholinergic anti-inflammatory pathway (as shown by higher RMSSD maternal values) (13, 21).

Efforts to alter emotional responses in emotionally arousing situations are associated with increased vagal activity (57). For example, women who suppressed or repressed their emotions reported substantial increases in baseline vagal activity relative to women who were not told to control their emotions (58). Theoretical models of self-regulation and parasympathetic functioning indicate that vagal activity increases when practicing self-regulation, leading to an increase in HRV (59). Anxiety during pregnancy has been associated with autonomic nervous system activity through frequency-domain indexes of HRV (60). In the present study, a significant reduction in the RMSSD by warm showering was observed, which may be seen as a better preparation for a higher precompetitive anxiety level, as previously noted (61). Thus, the reduction of RMSSD by warm shower therapy could be interpreted as a change of sympathovagal balance as an optimal anticipation of stress higher levels toward a shift toward sympathetic predominance as a result of parasympathetic withdrawal (61). However, due to the study design, the results of this study cannot provide evidence that the warm shower increased sympathetic modulation. Previous research, including in pregnant women, has suggested that a correlated pattern of high resting vagal tone combined with rapid reactivity and recovery represents “vagal flexibility,” or the ability to calibrate physiological stress systems in response to situational demands (62, 63). As in previous studies, hydrotherapy reduced anxiety in this study, promoting relaxation during labor (5), hence reducing the likelihood of dystocia (64). Hydrotherapy also promotes spontaneous labor without analgesia, which, according to prior research, appears to improve cardiovagal modulation and postnatal cardiorespiratory adaption in newborns (65). Based on these findings, a main benefit of hydrotherapy appears to be that it reduces anxiety while preventing parasympathetic modulation from decreasing to the same extent as observed in the IV ANALG group.

This study also provides evidence on the effects of intravenous analgesia (scopolamine + sodium dipyrone) or spinal anesthesia (bupivacaine + morphine) on HRV. This study to investigated the effects of intravenous analgesia with scopolamine + sodium dipyrone on HRV, while most of research studied the HRV effects by transdermal scopolamine. Scopolamine is an anticholinergic compound, so it stands to reason that it may affect parasympathetic modulation. Indeed, RMSSD, which reflects primarily parasympathetic activity, was substantially reduced. This drop in RMSSD was accompanied by a significant decrease in the E/I ratio, further substantiating a reduction in parasympathetic activity by intravenous analgesia (scopolamine + sodium dipyrone) (66). Furthermore, the overall HRV (SDNN, CV) decreased. The rise in the mean heart rate indicates a transition to sympathetic modulation. In other studies, only low-dose scopolamine (transdermal 0.5–1.5 mg) has been tested and shown to reduce heart rate through a paradoxical vagomimetic effect while increasing cardiac parasympathetic activity in healthy subjects (67, 68). Spinal anesthesia did not alter cardiac parasympathetic modulation (RMSSD) or overall HRV (SDNN, CV), while it reduced the mean heart rate. These findings support previous findings that labor epidural analgesia has no significant impact on autonomic heart rate regulation (69). However, others have observed that labor epidural analgesia (bupivacaine with fentanyl) induces somatosensory blockade and changes in autonomic outflow, while HR variability was shown to be a surrogate marker of increased parasympathetic activity (70).

Deep breathing exercises have been used to reduce anxiety and to increase parasympathetic activity assessed by HRV indexes (71). In addition, there is increasing evidence that slow deep breathing (SDB) may help patients with acute pain (72). Two studies involving 390 patients with acute obstetric labor pain showed that the use of breathing patterns during the first stage of labor were ineffective in reducing anxiety and pain (73), but were effective in reducing the perception of labor pain and shortening the duration of the second stage of delivery (74). Moreover, the breathing technique of blowing can be a viable alternative to the Valsalva maneuver in order to reduce perineal damage in laboring women (75). Assessing cardiac autonomic modulation during deep breathing exercises will provide further insight into the involvement of the autonomic nervous system during labor and delivery.

### Limitations of the study

Possible influences on autonomic nervous system activity during partum by variables such as tidal volume, level of cervical dilation, anxiety and pain levels, as well as maternal age, pregnancy age, level of education, parity, or associated pathologies could not be tested, and thus more research is needed to investigate and identify such variations. Furthermore, maternal variables of postpartum anxiety or depression and measures of optimism on any scale were not collected. In addition, due to the nature of the study, single- or double-blinding was not possible; therefore, we attempted to reduce any potential bias in the study protocol by having the statistician remain blinded to the coding allocation until the conclusion of the analysis. The lack of a negative control group (i.e., natural birth without intervention) prevents drawing definitive conclusions, as the decrease in parasympathetic indices occurred in both hydrotherapy (HydroTx) and intravenous analgesia (IV ANALG), but not in spinal anesthesia (Spinal ANES).

### Clinical implications

The dependability of hydrotherapy as a non-pharmacological strategy is linked to the completion of further clinical trials demonstrating quantitative evidence through outcome biomarkers to support indications relating to stress and birth development. In this way, hydrotherapy may contribute to family-based ambulatory treatment in the first and beginning of the second stages of labor (ambulatory in many settings and countries) and efforts to promote either natural birth or the judicious minimization of obstetric procedures that pose increased health risks. These are essential steps to resolve the dynamic challenges of delivering equal, healthy, and cost-effective maternal services responsive to all participants and studies in the health sciences.

The development of reliable autonomic nervous system assays to be used as point-of-care tests to supplement clinical decision-making is of current interest (31). Emerging biotechnologies for assessing the disruption of the autonomic nervous system caused by stress or disease involve research into HRV, electrodermal activity, and sweat sensing, pupillary light reflex, and aptamer-based detection of salivary biomarkers for discovery and development of biomarkers at the point-of-care setting (31, 76–78). In this study, the efficient use of a HRV deep breathing test during the first stage of labor performed with a miniaturized analytical device demonstrates that such testing could be used as a point-of-care in various clinical scenarios.

## Conclusion

These findings support the use of warm hydrotherapy as a non-pharmacological method to modulate sympathovagal balance and reduce overall labor anxiety during the first stage of labor.

## Data availability statement

The datasets generated during and/or analyzed during the current study are available from the corresponding authors on reasonable request.

## Ethics statement

The protocol was approved by Anhembi Morumbi University Ethical Committee (CAAE: 15787719.50000.5492). The patients/participants provided their written informed consent to participate in this study.

## Author contributions

LC, OB, and RD: study conception and design. RD and CF: perform the study. RD, CF, DN, and LC: assays and data analysis. LC, OB, JA, RG, and JS: interpretation of the results. LC, OB, JA, and JS: writing of the manuscript. RG, RD, CF, DN, JS, JA, OB, LC, and OB: critical revision of the manuscript regarding the important intellectual content. All authors contributed to the article and approved the submitted version.

## Footnotes

1
www.graphpad.com
